# An HPLC-UV Method to Assess Human Plasma 25(OH)D_3_

**DOI:** 10.3390/nu16142304

**Published:** 2024-07-18

**Authors:** Alexandra Tijerina, Aurora Garza, Abad López, Norma Cavazos, Ana Romo, Michel S. Heya, Cristina Bouzas, Josep A. Tur, Rogelio Salas

**Affiliations:** 1 Faculty of Public Health and Nutrition, Autonomous University of Nuevo Leon, Monterrey 64460, NL, Mexico; 2 Faculty of Medicine, Autonomous University of Nuevo Leon, Monterrey 64460, NL, Mexico; 3 Research Group on Community Nutrition and Oxidative Stress, University of Balearic Islands—IUNICS, IDISBA & CIBEROBN, Guillem Colom Bldg, Campus, 07122 Palma de Mallorca, Spain; 4 Health Institute of the Balearic Islands (IDISBA), 07120 Palma de Mallorca, Spain; 5 CIBER Physiopathology of Obesity and Nutrition (CIBEROBN), Institute of Health Carlos III (ISCIII), 28029 Madrid, Spain

**Keywords:** 25-hydroxyvitamin D_3_, HPLC-UV, linearity, precision, robustness, accuracy

## Abstract

The aim of this study was to validate an HPLC-UV method to assess vitamin D status by determining the linearity and precision of the 25-hydroxyvitamin D_3_ (25(OH)D_3_) calibration curve, the limits of detection, quantitation and robustness of the method, and its accuracy. A second stock solution of 25(OH)D_3_ was prepared (500 ng/mL), and working dilutions (5, 10, 20, 30, 40, and 50 ng/mL) were prepared for a calibration curve. The HPLC equipment had a UV-Vis diode-array detector and utilized an Acclaim^TM^ 120 C18 column (5 µm, 4.6 × 250 mm) with a flow rate of 1.2 mL/min, a column temperature of 30 °C, and the standards and samples were maintained at 4 °C, with an injection volume of 100 µL. Detection of 25(OH)D_3_ was determined at 265 nm, with a retention time of 4.0 min. The validation was conducted according to the FDA Validation of Analytical Procedures: Guidance for Industry. Vitamin D was extracted from plasma samples using acetonitrile (ACN)–0.1% formic acid (2:1 *v*/*v*), and the percentage of recovery was calculated. The proposed method conditions gave excellent linearity (R^2^ = 0.9989) and the linearity coefficient was R^2^ > 0.99 for 25(OH)D_3_. The detection and quantification limits were 1.1703 ng/mL and 3.5462 ng/mL, respectively. Decreasing or increasing the reading temperature by 1 °C decreased the response units (AU) of vitamin D, 25(OH)D_3_. When the current flow rate decreased by 0.2 mL/min (1.0 mL/min), the retention time increased to 4.913 min, whereas an increase of 0.2 mL/min of the proposed flow rate (1.4 mL/min) decreased the retention time to 3.500 min. The percentage of recovery varied from 92.2% to 97.1%. The proposed method to quantify a vitamin D metabolite (25(OH)D_3_) in human plasma samples was reliable and validated.

## 1. Introduction

Vitamin D is a liposoluble vitamin that acts as a pro-hormone [1]. It is found in two bioequivalent forms: ergocalciferol (D_2_), which is acquired from vegetable sources and oral supplements, and cholecalciferol (D_3_), obtained through biosynthesis in the skin via solar exposure to ultraviolet energy, from the diet (especially from animal origin), and oral supplements [2,3]. In the liver, D_2_ and D_3_ are metabolized by hydroxylation, resulting in 25-hydroxyvitamin D (25(OH)D) [4]. A second hydroxylation mostly occurs in the kidney, forming 1α,25-dihydroxyvitamin D (1,25(OH)_2_D), which is known as the active form of vitamin D [2,5]. Other tissues that convert 25(OH)D to 1,25(OH)_2_D include the brain, uterus, placenta, and vascular smooth muscle cells [6,7].

The concentration of 25-hydroxyvitamin D_3_ (25(OH)D_3_) is the most frequently used biomarker to measure vitamin D status [2,5,8,9,10,11,12,13], since it is available in higher concentrations [14] and represents both forms of vitamin D from dietary sources, supplementation, and solar exposure [9].

Different analytical methods are available to quantify vitamin D in human plasma or serum samples, such as high-pressure liquid chromatography (HPLC), enzyme-linked immunoassay (ELISA) [15], liquid chromatography with mass spectrometry (LC-MS/MS), radioimmunoassay (RIA), CREB-binding protein (CBP) assay, and the chemiluminescence immunoassay (CLIA) [16].

Some advantages of the HPLC method to quantify vitamin D status include its low bias and variability, the capability to separately measure D_2_ and D_3_ metabolites, and lower reagent costs compared to immunoassays. Liquid chromatography with mass spectrometry (LC-MS/MS) was suggested as the gold standard method to assess vitamin D status [6]; however, this equipment is expensive and is not fully available in all laboratories. Immunoassays, such as RIA and ELISA, are highly variable, underscoring the need for standardized laboratory techniques worldwide [5]. Immunoassays are susceptible to cross-reactivity with vitamin D metabolites, such as 24,25-dihydroxyvitamin D (24,25(OH)_2_D) [17]. Although, a novel chemiluminescence immunoassay with high selectivity and stability for 25(OH)D in human serum samples has recently been reported [18].

In HPLC methodologies, sample preparation prior to biomarker analysis is of relevance for obtaining a better chromatogram image, thereby improving the accuracy of calculations and the interpretation of results. For biological samples, such as plasma or serum, the main steps include protein precipitation, concentration by drying, and reconstitution [19,20,21]. Different methodologies for metabolite extraction have been proposed, using single reagents such as methanol [19], simple mixes such as ethanol–acetonitrile (2:1, *v*/*v*) [2], or more complex mixes such as acetonitrile–methanol–0.1% formic acid (60:20:20 (*v*/*v*)) [20], resulting in a wide range of metabolite recovery percentages.

The aim of this study was to standardize and validate a simple HPLC-UV method to assess vitamin D status. This involved determining the linearity and precision of a 25(OH)D_3_ calibration curve, as well as establishing the limits of detection and quantitation, and assessing the robustness of the method. The accuracy of the method to assess vitamin 25(OH)D_3_ concentration in plasma samples was also calculated. The proposed method also aimed to optimize sample preparation to achieve greater recovery results, thereby facilitating easier identification and quantitation of the 25(OH)D_3_ metabolite.

## 2. Methods

### 2.1. Chemicals and Equipment

Standard 25-hydroxyvitamin D_3_ (25-hydroxycholecalciferol; 25(OH)D_3_) (Sigma Aldrich, St. Louis, MO, USA, HPLC grade), methanol (MeOH) (TEDIA, Fairfield, OH, USA, HPLC grade), acetonitrile (ACN) (TEDIA, Fairfield, OH, USA, HPLC grade), ethanol (EtOH) (TEDIA, Fairfield, OH, USA, HPLC grade), formic acid (Sigma-Aldrich, St. Louis, MO, USA, HPLC grade), and milli-Q^®^ water (prepared in Ultrapure (type 1) filtration equipment, Simplicity^®^ UV, Merck KGaA, Darmstadt, Germany) were used.

A Centrivap concentrator (Labconco Corporation, Fort Scott, KS, USA), a centrifuge Solbat J-40 (Solbat, Puebla, Mexico), an Eppendorf^®^ Microcentrifuge minispin Plus (Eppendorf, Hamburg, Germany), and HPLC equipment (Waters Alliance e2695, with Waters Empower™ 3 software, Waters Corp., Milford, MA, USA) were used.

### 2.2. Vitamin D Standard, Calibration Curve, and Blank

A standard solution of 25(OH)D_3_ with ACN was prepared (1 mg/mL). From this, a stock solution was prepared (5000 ng/mL), followed by several second stock solutions (500 ng/mL) prepared in ACN. All stock solutions were covered in aluminum foil and kept at −80 °C until use. From the second stock solutions, working dilutions (5, 10, 20, 30, 40, and 50 ng/mL) were prepared in ACN and protected from light.

### 2.3. HPLC Equipment

The HPLC equipment included a UV-Vis diode-array detector (Waters Alliance e2695), a mobile phase reservoir, vacuum degas system, a front panel control configured in carousels for up to 120 vials, automatic sample management, and a heater and cooler for the samples and the columns. The Waters Empower™ 3 software (Waters Corp., Milford, MA, USA) was used to control, process, and obtain data.

### 2.4. Chromatographic Conditions

The column used was an Acclaim^TM^ 120 C18 (5 µm, 4.6 × 250 mm) (Acclaim, Glen Cove, NY, USA). The mobile phase was MeOH-ACN (80:20, *v*/*v*) with an isocratic elution, similar to previously reported methodology [19], with a flow rate of 1.2 mL/min. The column temperature was set at 30 °C, and the standards and samples were kept at 4 °C. The injection volume was 100 µL. The run time was set at 25 min per sample, with column washing between samples from minute 13 to 17 with acidified milli-Q^®^ water and from minute 18 to 25 with the mobile phase. This ensured thorough washing of the column to remove the attached plasma components and achieve system equilibrium before analyzing the next sample. Detection of 25(OH)D_3_ was found to be optimal at an absorbance wavelength of 265 nm and with a retention time of 4.0 min.

All extractions, standard, dilutions, sample preparations, and measurements were conducted in darkness and the tubes were carefully protected from light.

### 2.5. Validation of the Chromatographic Method

Validation of the chromatographic method was conducted according to the FDA Validation of Analytical Procedures: Guidance for Industry [22]. The parameters evaluated included linearity, detection and quantitation limits, precision, robustness, and accuracy.

### 2.6. Linearity

Linearity was determined by plotting a 6-point calibration curve (5, 10, 20, 30, 40, and 50 ng/mL) of 25(OH)D_3_, according to the linear regression equation and the determination coefficient (R^2^). The calibration curve was run across three different days, and the average area of each point was calculated and plotted against its concentration. The linear regression equation and R^2^ of the calibration curve were calculated in Excel (Microsoft Office 2013, Microsoft, Albuquerque, NM, USA).

### 2.7. Detection and Quantitation Limits

The analysis of limits was based on the standard deviation of the linear response and the slope. The detection limit (DL) was determined as 3.3 times the standard deviation, and the quantitation limit (QL) was 10 times the standard deviation of the response, as follows:

DL = 3.3 SD/m       QL = 10 SD/m

where SD represents the standard deviation of the response and m represents the slope of the calibration curve.

The standard deviation (SD) of the response was calculated from 10 runs of the blank, and the slope (m) was obtained from the linear equation of the calibration curve in the linearity test. The limits were then calculated in ng/mL based on the equation of the calibration curve.

### 2.8. Precision and Repeatability

To determine the repeatability of the method, the percentage of variance (%CV) (or relative standard deviation, RSD) was calculated from the calibration curve (5–50 ng/mL) of 25(OH)D_3_. Each data point was measured in triplicate.

Intermediate precision was calculated as the percentage of variance (%CV) using three selected concentrations from the calibration curve (low: 5 ng/mL, moderate: 30 ng/mL, and high: 50 ng/mL). Each selected point was measured in triplicate over three different days, and a new 25(OH)D_3_ stock solution was prepared each day.

### 2.9. Robustness

The robustness of the method was determined by analyzing significant changes in HPLC areas while changing the column temperature and the flow rate. A 25(OH)D_3_ solution of 50 ng/mL was used, and the following combinations were studied in triplicate: 29 °C and 1.2 mL/min, 31 °C and 1.2 mL/min, 30 °C and 1 mL/min, and 30 °C and 1.4 mL/min.

The proposed equation [18], as subsequently explained [23], was used to determine robustness:

*| Vx |* > SDx × √2

where *| Vx |* is ¼ of the selected 25(OH)D_3_ concentration of 50 ng/mL with changes in parameters minus ¼ of the concentration with the established parameters. SDx represents the standard deviation of the repeatability test, where √2 denotes the square root of 2.

### 2.10. Accuracy

The accuracy of the method was determined as the % of recovery after extracting vitamin D from fortified plasma samples.

### 2.11. Plasma Samples

The samples were obtained in the summer of 2022 from 40–60-year-old healthy women (n = 10) living in metropolitan areas of Monterrey, Nuevo León, Mexico. Blood was extracted after a 12-h overnight fast into suitable tubes from the antecubital vein (EDTA-K tubes). Then, the plasma was obtained by centrifugation at 3500 rpm for 12 min. The plasma samples were frozen at −80 °C until use. It is recommended to store samples at −80 °C (the method of choice for freezing samples) for up to 12 months (conservative), or at −20 °C for up to 4 weeks, following standard recommendations. Appropriate sample handling procedures were followed, including protection from light, and the stability of the metabolite was considered based on other studies [24,25,26].

### 2.12. Extraction of Vitamin D from Plasma Samples

Adapted from previous studies [2,19,20], a total of 500 μL of plasma (non-fortified and fortified) was mixed with 1000 μL of each solvent for vitamin D extraction (Table 1) and left for 3 min. The extraction of vitamin D followed methods defined in previous studies [2,20] and, as proposed in this study, was as follows:

The plasma samples were vortexed to precipitate proteins for 30 s, followed by micro-centrifugation for 15 min at 3000× *g*. The upper layer was collected and vacuum dried for 4 h at 30 °C. The dry samples were stored at −20 °C for 20 h before reconstitution.

A volume of 250 μL of the extraction solvent was added to the dry sample and vortexed for 30 s. The reconstituted samples were filtered using a 13 mm syringe filter with a pore diameter of 0.45 µm. They were then transferred into glass inserts within amber vials and analyzed using HPLC-UV.

### 2.13. Percentage of Recovery

The concentration of 25(OH)D_3_ in fortified plasma samples (C_F_) against non-fortified plasma sample (C_NF_) was divided by the theoretical concentration of 25(OH)D_3_ (C_T_ = 40 ng/mL) and multiplied by 100 to report the result as a percentage (%), as follows:

$$\%\;\mathrm{Recovery}\;=\;\frac{C_{F}\;-\;C_{\mathrm{NF}}\;}{C_{T}}\;\times \;100$$

where C_F_ represents the concentration of 25(OH)D_3_ in the fortified plasma sample, C_NF_ represents the concentration of 25(OH)D_3_ in a non-fortified plasma sample, and C_T_ represents the theoretical concentration of 25(OH)D_3_ added to the fortified sample (40 ng/mL). Recovery was conducted in triplicate using three different human plasma samples.

### 2.14. Plasma Levels of 25(OH)D_3_

The level of 25(OH)D_3_ in the sample was calculated by analyzing the concentration in the fortified plasma (10 ng/mL), as follows:

$$\mathrm{Concentration}\;\mathrm{of}\;25(\mathrm{OH})D_{3}=\;\frac{\left(C_{F}\;-\;C_{T}\right)\;\times \;d.f.\;\times \;100}{\%\;\mathrm{recovery}}$$

where C_F_ represents the concentration of 25(OH)D_3_ in the fortified plasma sample, C_T_ represents the theoretical concentration of 25(OH)D_3_ added to the fortified sample (10 ng/mL), % recovery denotes the average recovery obtained from the accuracy assay, and d.f. denotes the dilution factor due to extraction, reconstitution, and injection.

### 2.15. Ethics and Laboratory Biosafety

This experimental protocol adhered to the guidelines outlined in the Declaration of Helsinki and underwent a thorough review and approval process by the Ethics Committee of the Faculty of Public Health and Nutrition (Reference: 21-FaSPyN-SA-19.TP; 30 September 2021). The participants were properly informed of the study aims, risks, and benefits and provided signed informed consent. The work conducted in the laboratory and the handling of biological samples, chemicals, and residues (chemical and infectious waste) followed the processes outlined in NOM-087-ECOL-SSA1-2002 [27] and the guidelines from the Department of Biosafety of the Faculty of Public Health and Nutrition.

## 3. Results

The chromatographic conditions of the proposed method are detailed in Table 2. The repeatability, expressed as the coefficient of variation (%CV), for six calibration points performed in triplicate, was calculated to be below 6.8%.

**Figure 1:**
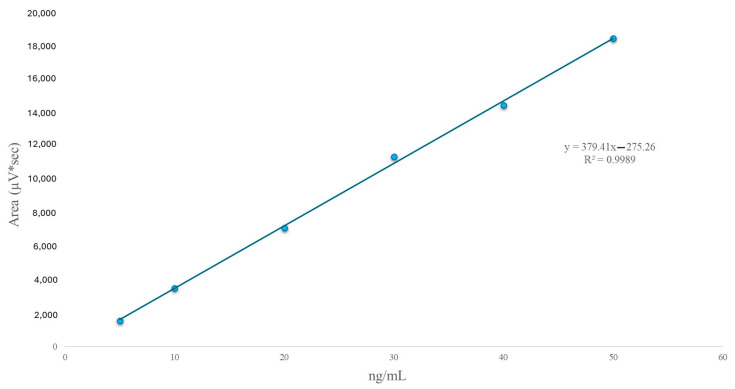
Calibration curve of 25(OH)D_3_ and the regression equation (R^2^ = 0.9989).

The calibration curve of 25(OH)D_3_ is shown in Figure 1. It resulted an R^2^ value of 0.9989, and the linear regression equation was y = 379.41x − 275.26.

**Figure 2:**
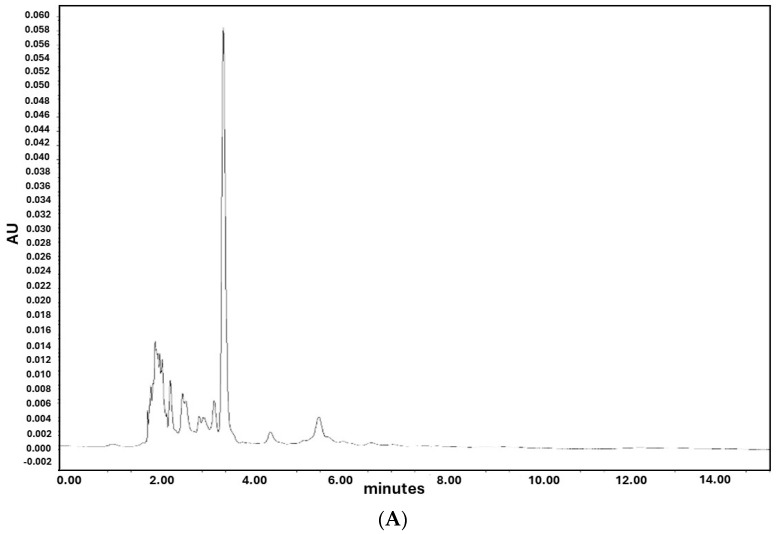

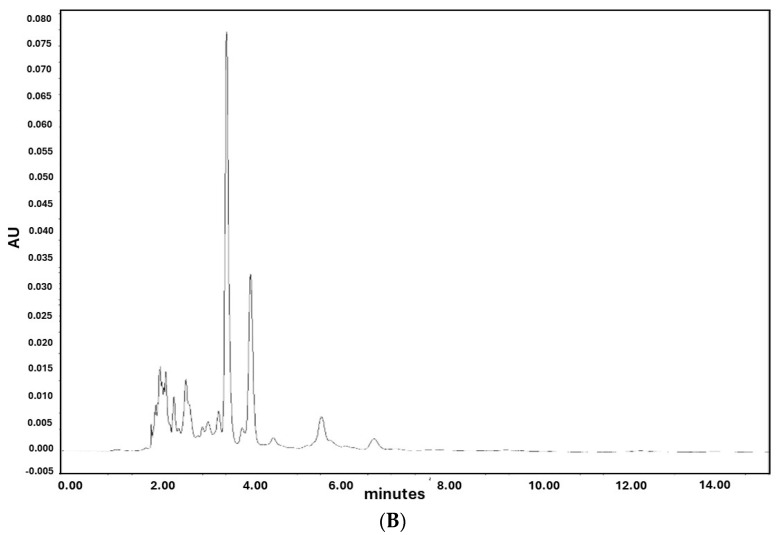
Chromatograms of a blank plasma sample (**A**) and a plasma sample spiked with 50 ng/dL of 25(OH)D_3_ (**B**). Detection is seen at minute 4.0. in (**B**).

The chromatograms of an example of a plasma sample are shown in Figure 2. Figure 2A depicts a blank plasma sample, whereas Figure 2B shows a sample spiked with the metabolite 25(OH)D_3_, detected at 4.0 min. The figures demonstrate no interference with other metabolites or plasma components.

Based on the standard deviation of a linear response and the slope, the detection limit (DL) was 1.1703 ng/mL, whereas the quantitation limit (QL) was 3.5462 ng/mL (Table 3).

Table 4 shows the results of intermediate precision. The average area (AU) increased with the calibration point, whereas the %CV was higher at the moderate calibration point and lower at the low calibration point.

Table 5 shows the average results of the modified conditions used to determine the robustness of the HPLC-UV method. The highest values of retention time (Rt) were recorded at 30 °C and 1 mL/min.

Table 6 shows the analysis of results based on the changes in HPLC conditions proposed for method robustness. The highest retention times were registered under condition 3.

Table 7 shows the results of the accuracy test, with an average recovery of 94.4% after plasma extraction.

Table 8 compares the calculated recovery of previous methods with that proposed in this current study. The use of acetonitrile and 0.1% formic acid (2:1 *v*/*v*) provided higher 25(OH)D_3_ recovery.

## 4. Discussion

The need for new methods to quantify plasma metabolites, such as vitamin D, has led to increased equipment costs and training requirements, together with the high variability in laboratory techniques. In the current study, a simple chromatographic method (HPLC) was standardized and validated to quantify 25(OH)D_3_ in plasma samples, following the FDA Validation of Analytical Procedures: Guidance for Industry [22].

The proposed method conditions were validated at 265 nm, with a retention time of 4.0 min, demonstrating excellent linearity and a coefficient of determination (R^2^) of 0.9989. This result is consistent with the literature, which suggests a linearity coefficient of R^2^ > 0.99 for this metabolite [2,21,28]. The detection and quantitation limits of 25(OH)D_3_ were 1.1703 and 3.5462 ng/mL, respectively, similar to findings from a previous Canadian study reporting a detection limit of approximately 2.0 ng/mL [21]. A calibration point of 5 ng/mL was determined as the lowest calibration point to ensure 25(OH)D_3_ quantitation in plasma samples.

The precision of the method was calculated to be below 6.8%, and the intermediate precision was below 7%, which is above the acceptable level of ≤ 2% for industry purposes [22]. Previous authors have reported HPLC methods for determining vitamin D with higher variances, up to 13.8% [11] and 15.1% [29], although these studies utilized more sophisticated technology, such as HPLC-MS/MS. Moreover, in the field of research and development, a precision below 10% is considered appropriate, making this appropriate for exploratory studies.

The robustness of the proposed method was also confirmed as chromatographic variations caused a negative effect on the chromatographic response with minor changes in method conditions, such as changes to the temperature and flow rate. The response was directly demonstrated on the product or suitable reference materials, with separate weightings of the analyte or predefined mixtures of the components (e.g., by dilution of a solution of known content). Decreasing or increasing the reading temperature by 1 °C decreased the response units (AU) of vitamin D, 25(OH)D_3_. Also, when the current flow rate decreased by 0.2 mL/min (1.0 mL/min), the retention time increased to 4.913 min, whereas an increase of 0.2 mL/min in the proposed flow rate (1.4 mL/min) decreased the retention time to 3.500 min. Flow rates between 1.0 and 1.5 mL/min were proposed as suggested by previous authors to reduce total measurement times [19]. In the current validation, the established flow rate and temperature conditions (1.2 mL/min and 30 °C) were also maintained since the 25(OH)D_3_ peak was not interfered with by any other metabolite in the plasma samples.

The donated plasma samples (*n* = 10) contained 25(OH)D_3_ levels between <5 and 31.8 ng/mL, with an average of 18.6 ng/mL, similar to the value reported in 46 healthy female volunteers from Venezuela (aged 50–94 years) of 19.74 ± 9.48 ng/mL [2]. Further research on 25(OH)D_3_ status in women aged 40–60 years could be of interest.

### Strengths and Limitations

Method accuracy could be affected by several factors, such as the extraction solvent used, the extraction method employed, the use of liquid–liquid and solid–liquid extraction systems, centrifugal force, and other variables. The main strength of this method is that the current recovery results ranged from 92.2 to 97.1%, demonstrating very good accuracy. Previous studies reported the accuracy of HPLC methods ranging from 89.6 to 97.1% [2,20]. In this current validation study, the best extraction solvent was acetonitrile and 0.1% of formic acid (2:1 *v*/*v*). The accuracy of the method using previously reported solvents [2,20] was also calculated. The current results demonstrate that using ACN and formic acid as the extraction solvent allows for a better percentage recovery of 25(OH)D_3_ from human plasma samples. This suggests efficacy in reducing possible noise, especially from proteins, which could negatively affect the detection and quantitation of the studied metabolite.

A major limitation of the proposed method is that an internal standard was not used for quality control purposes; however, the use of spiked samples and recovery assessment with 25(OH)D_3_ resulted in very good accuracy for the quantitation of human plasma samples. In addition, the proposed method has obtained results comparable to those previously reported [2,9,10,11,12,19,20,21], suggesting that ours could serve as an alternate HPLC method demonstrating accuracy and reliability. Another limitation of the proposed method is the use of chemicals and their residues, which may affect the environment. Therefore, proper management practices were followed according to the institution’s Biosafety Department, based on Mexican standards for environmental protection [27].

## 5. Conclusions

The proposed method for quantifying a vitamin D metabolite (25(OH)D_3_) in human plasma samples was reliable and complied with the validation criteria for linearity, precision, accuracy, and robustness, as required for the standardization of HPLC methodologies. Measuring vitamin D in human plasma samples will help to understand nutritional status in population settings.

## Figures and Tables

**Table 1:** Solvents for vitamin D extraction from plasma samples.

Method (Ref.)	Solvents for Extraction
Brunetto et al., 2004 [2]	Ethanol–Acetonitrile (2:1 (*v*/*v*))
Mathew et al., 2019 [20]	Acetonitrile–Methanol–0.1% Formic acid (60:20:20 (*v*/*v*))
Proposed *	Acetonitrile–0.1% Formic acid (2:1 (*v*/*v*))

* Proposed in this current study.

**Table 2:** Repeatability results.

Calibration Point (ng/mL 25(OH)D_3_)	Average Area (AU)	SD	%CV
5	1552.93	46.33	2.98
10	3536.67	163.37	4.62
20	7208.17	365.66	5.07
30	11,514.40	781.37	6.79
40	14,638.83	836.27	5.71
50	18,705.67	555.33	2.97

Area: average area of triplicates. Abbreviations: SD, standard deviation; %CV, coefficient of variation.

**Table 3:** Detection and quantitation limits for 25(OH)D_3_.

Limit	Equation	Concentration (ng/mL)
Detection limit (DL)	DL = 3.3 SD/m	1.1703
Quantitation limit (QL)	QL = 10 SD/m	3.5462

SD: standard deviation, m: slope.

**Table 4:** Results of intermediate precision.

Calibration Point (ng/mL 25(OH)D_3_)	Average Area (AU)	SD	%CV
5 (low)	1555.78	41.72	2.68
30 (moderate)	10,942.80	759.07	6.94
50 (high)	18,713.90	653.93	3.49

Area: average area of nine measurements. Abbreviations: SD, standard deviation; %CV, coefficient of variation.

**Table 5:** Area and retention time after modifying the conditions.

Condition	Average Area (AU)	Rt (min)
29 °C—1.2 mL/min	16,541	4.121
2. 31 °C—1.2 mL/min	16,617	4.052
3. 30 °C—1.0 mL/min	19,218	4.913
4. 30 °C—1.4 mL/min	18,947	3.500

Rt: retention time.

**Table 6:** Results according to the changes in HPLC conditions for method robustness.

	Vx		SDx × √2		*| Vx |* > SDx × √2	
HPLC Conditions	Area (AU)	Rt (min)	Area (AU)	Rt (min)	Area (AU)	Rt (min)
condition 1 29 °C—1.2 mL/min	594.125 600.625 618.375	−0.019 −0.019 −0.018	180.408	0.0071	Yes	Yes
condition 2 31 °C—1.2 mL/min	579.875 581.625 594.875	−0.002 −0.000 −0.001	180.408	0.0071	Yes	No
condition 3 30 °C—1.0 mL/min	−65.375 −62.875 −66.125	−0.216 −0.217 −0.216	180.408	0.0071	No	Yes
condition 4 30 °C—1.4 mL/min	10.375 −5.625 3.625	0.137 0.138 0.137	180.408	0.0071	No	Yes

Rt: retention time, Vx: ¼ difference in calculated concentration, SDx: standard deviation of the repeatability test, *| Vx |*: absolute value of Vx. Yes denotes a significant change in the result parameters. No denotes no significant change in the result parameters.

**Table 7:** Recovery (%) of 25(OH)D_3_ from fortified plasma samples.

Plasma Sample	Concentration (ng/mL)	Recovery (%)
NF 1	85.9 ± 5.5	97.1 ± 5.4
F 1	124.7 ± 5.8	
NF 2	78.9 ± 10.2	92.2 ± 19.8
F 2	115.8 ± 3.7	
NF 3	58.9 ± 7.9	94.0 ± 27.7
F 3	96.5 ± 13.1	

NF: non-fortified plasma sample, F: plasma sample fortified with 25(OH)D_3_.

**Table 8:** Comparison of the recovery of 25(OH)D_3_ in previously reported methods and the extraction method proposed in this study.

Method (Ref.)	Solvent for Extraction	Calculated Recovery (%)
Brunetto et al., 2004 [2]	Ethanol—Acetonitrile (2:1 *v*/*v*)	40 *
Mathew et al., 2019 [20]	Acetonitrile—Methanol—0.1% Formic acid (60:20:20 *v*/*v*)	50–65 *
Proposed	Acetonitrile—0.1% Formic acid (2:1 *v*/*v*)	92.2–97.1

* Calculated recovery was determined in this current study.

## Data Availability

There are restrictions on the availability of data for this trial due to signed agreements governing data sharing. Access to the trial data is limited to external researchers conducting studies aligned with the project’s purposes. Requestors wishing to access the trial data used in this study can make a request to pep.tur@uib.es.
